# RLC Circuit Forecast in Analog IC Packaging and Testing by Machine Learning Techniques

**DOI:** 10.3390/mi13081305

**Published:** 2022-08-12

**Authors:** Jung-Pin Lai, Ying-Lei Lin, Ho-Chuan Lin, Chih-Yuan Shih, Yu-Po Wang, Ping-Feng Pai

**Affiliations:** 1 PhD Program in Strategy and Development of Emerging Industries, National Chi Nan University, Nantou 54561, Taiwan; 2 Siliconware Precision Industries Co., Ltd., No. 123, Sec. 3, Dafeng Rd., Dafeng Vil., Tanzi Dist., Taichung City 42749, Taiwan; 3 Department of Information Management, National Chi Nan University, Nantou 54561, Taiwan

**Keywords:** integrated circuit, packaging and testing, machine learning, LSSVR

## Abstract

For electronic products, printed circuit boards are employed to fix integrated circuits (ICs) and connect all ICs and electronic components. This allows for the smooth transmission of electronic signals among electronic components. Machine learning (ML) techniques are popular and employed in various fields. To capture the nonlinear data patterns and input–output electrical relationships of analog circuits, this study aims to employ ML techniques to improve operations from modeling to testing in the analog IC packaging and testing industry. The simulation calculation of the resistance, inductance, and capacitance of the pin count corresponding to the target electrical specification is a complex process. Tasks include converting a two-dimensional circuit into a three-dimensional one in simulation and modeling-buried structure operations. In this study, circuit datasets are employed for training the ML model to predict resistance (R), inductance (L), and capacitance (C). The least squares support vector regression (LSSVR) with Genetic Algorithms (GA) (LSSVR-GA) serves as an ML model for forecasting RLC values. Genetic algorithms are used to select parameters of LSSVR models. To demonstrate the performance of LSSVR models in forecasting RLC values, three other ML models with genetic algorithms, including backpropagation neural networks (BPNN-GA), random forest (RF-GA), and eXtreme gradient boosting (XGBoost-GA), were employed to cope with the same data. Numerical results illustrated that the LSSVR-GA outperformed the three other forecasting models by around 14.84% averagely in terms of mean absolute percentage error (MAPE), weighted absolute percent error measure (WAPE), and normalized mean absolute error (NMAE). This study collected data from an IC packaging and testing firm in Taiwan. The innovation and advantage of the proposed method is using a machine approach to forecast RLC values instead of through simulation ways, which generates accurate results. Numerical results revealed that the developed ML model is effective and efficient in RLC circuit forecasting for the analog IC packaging and testing industry.

## 1. Introduction

Circuit simulation work in the integrated circuit (IC) packaging process depends on model complexity in geometry and electromagnetic materials. Properly simulating IC packaging plays a vital role in catching potential EMC, power, and signal integrity issues early in the design process and overcoming major pitfalls. The elimination of manual processing is required to reduce the time and effort from customer requirement specifications to IC packaging design, testing, manufacturing, and to determine the tools and process options for application design. Implementation takes prediction, evaluation, and decision making with machine learning-centric databases, tools, and design models. Learning-based tools and process models must continuously improve through additional design experience [1,2].

Machine learning (ML) solved many problems that were originally difficult to solve in data science. Many studies have shown that machine learning and optimization algorithms are suitable for solving different problems in the IC packaging and design processes, reducing design errors and design cycle time [3]. Table 1 shows the latest research on the IC packaging process using machine learning.

The signal passes through the substrate of the printed circuit board. Simulation provides the designer with a pre-optimized design concept. Ren et al. [4] introduced a graph neural network to predict network parasitics and device parameters by converting circuit schematics into graphs and utilizing key GNN-based modeling techniques. The results showed that the average simulation error was reduced from over 100% and estimated by designers to be less than 10%. Shook et al. [5] proposed a new machine learning-based parasitic estimation method for pre-layout custom circuit designs. For various analog circuits, the results show a reduction in the average error between pre-layout and post-layout circuit simulations from 37% to 8%.

For optimization and evaluation of package structural characteristics, Wu and Chu [6] proposed and verified an analog-driven design method for chip package integration structure design optimization. The study’s results suggest that the random forest algorithm can predict stress for chip package-integrated design. Hsiao and Chiang [7] proposed applying the RF model to predict the reliability of wafer-level packaging. The designers can easily optimize the WLP structure and shorten the design cycle. Lee et al. [8] developed a chip-to-package interactive risk assessment platform using finite element analysis, meta-modeling, and genetic algorithm optimization methods.

Heat transfer analysis of package structures is important in package functional testing. Acharya et al. [9] used three ML algorithms, random forest, support vector regression, and a neural network to model thermal behavior through hotspot temperature simulation data evaluation. They proposed an ML-based thermal design method and provided a reference frame for future packaging materials. Durgam et al. [10] used several machine learning methods to predict the temperature of the heat source on the substrate. The results showed that the temperature agreement between the prediction and the simulation was less than 10%. Jing et al. [11] proposed using the genetic algorithm to optimize the temperature curve prediction model in the reflow soldering process. The results show that the predicted value meets the error accuracy requirements. The results also prove that the established mathematical model can effectively predict temperature curves.

The power delivery network (PDN) must reliably supply power to functional blocks in an integrated circuit (IC). A robust PDN design has always been a critical challenge. Cecchetti et al. [12] developed a Genetic Algorithm (GA) and Artificial Neural Network (ANN) model for iterative optimization of the placement of decoupling capacitors in a PDN. They concluded that the GA-ANN model is consistent with the results of commercial simulator optimization. Sourav et al. [13] presented an ML architecture that combined neural networks and regression trees to predict printed circuit board (PCB) inductance and resistance. They employed an LSTM model to predict voltage drop as a function of time. The average prediction accuracy of the proposed method is 94%.

There have been many models utilizing machine learning and optimization to solve issues in IC packaging. Mao et al. [14] proposed a machine learning (ML) model based on the backpropagation (BP) method for predicting three-dimensional board-level drop responses for ball grid array (BGA) encapsulation structures. Jin et al. [15] constructed several machine learning methods to accurately predict the radiated electric field of wire-bonded ball grid array packages. They optimized model parameters to minimize the prediction error of each model. Their conclusion shows that DNN is an effective and feasible prediction model. Wang et al. [16] proposed a reverse design method based on convolutional neural networks for the fast optimization and design of encapsulation structures. Schierholz et al. [2] provided a database that allows for the study of machine learning tools and techniques in signal integrity, power integrity, and electromagnetic compatibility. It contains printed circuit board (PCB)-based interconnects and physics (PB). The corresponding frequency domain data of the tool can be used for different types of structural simulations.

**Table 1:** Summary of ML-based IC packaging process applications.

Literature	Years	Application	Method(s)
Ren et al. [4]	2021	Predict net parasitics and device parameters	GNN
Shook et al. [5]	2020	Parasitic estimation	Random forest
Wu and Chu [6]	2021	The structural design optimization of chip package integration	Random forest
Hsiao and Chiang [7]	2020	Packaging reliability analysis and prediction	Random forest
Lee et al. [8]	2021	Interactive risk assessment of chip packaging	FEA, MOGA
Acharya et al. [9]	2021	Predict the thermal behavior of a power electronics package	Random forest, SVR, ANN
Durgam et al. [10]	2022	The optimization of temperature on printed circuit board	XG Boost, ANN, SVR, RFR
Jing et al. [11]	2021	Predicting the temperature curve of SMT reflow soldering	Genetic Algorithm
Cecchetti et al. [12]	2020	Power delivery network (PDN)	ANN, Genetic Algorithm
Sourav et al. [13]	2020	Power delivery network (PDN)	Regressor trees, LSTM
Mao et al. [14]	2022	Predicting three-dimensional board-level drop responses for ball grid array (BGA) encapsulation structures	BPNN
Jin et al. [15]	2022	Predicting the radiated electric field of a wire-bonded ball grid array package	DNN, SVR, K-nearest neighbors, LR
Wang et al. [16]	2021	Full wave radiation simulation of package design process	CNN
Schierholz et al. [2]	2021	Signal integrity (SI) and power integrity (PI) database based on PCB interconnection	ANN, Genetic Algorithm

This study attempted to use least squares support vector regression with genetic algorithms to predict the RLC (resistance, inductance, and capacitance) values currently generated by simulation methods. The genetic algorithms were employed to determine LSSVR parameters to improve forecasting accuracy. The designed method employs a machine learning approach to forecast RLC values instead of through simulation ways and generates more accurate results than the three other machine learning models. The rest of this study is organized as follows. Section 2 provides the substrate and interface electrical transfer properties based on IC packages. Section 3 briefs the LSSVR model and genetic algorithms. The flowchart of the LSSVR-GA model for predicting RLC values is also addressed and presented. Numerical results are illustrated in Section 4. Conclusions are indicated in Section 5.

## 2. The Substrate and Interface of the IC Package Transmit Electrical Properties

**Figure 1:**
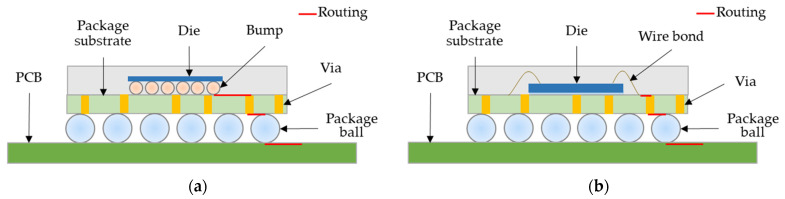
IC Package connections with substrate [19]. (**a**) Flip chip; (**b**) wire bonding.

In IC packaging design, the substrate is used as a carrier. The functions of the substrate are to protect and carry the IC chip and serve as a medium for circuit signal transmission. Integrated circuit packaging is the final stage of semiconductor component manufacturing. As a method for connecting the die to the external circuit, the chip’s packaging considers the pin configuration, electrical performance, heat dissipation, and the chip’s physical size. There are many typical packaging forms in the semiconductor industry [17,18]. Currently, the most common internal packaging methods of integrated circuits are wire bonding (WB) and flip chip (FC) packages. Flip chip packaging connects the chip to the bump and then turns the IC chip over to directly connect the bump and substrate. The wire bonding package places the chip on the substrate (chip pad) and then uses the wire bonding technology to connect the chip to the connection point on the substrate. The IC substrate acts as a buffer interface for electrical connection and transmission between the IC die and the PCB through the conductive routing and vias (VIA) network, as shown in Figure 1.

The RLC circuit is essential to evaluate the overall interface transmission capability in the IC packaging design process. It is a circuit structure composed of resistors, capacitors, and inductors. Parasitic effects associated with ICs and printed circuit board (PCB) conductors and their paths are essential parameters of the electrical transport model. The parasitic effects of RLC lines in the IC package process can cause signal integrity problems due to signal attenuation and delay [20,21].

**Figure 2:**
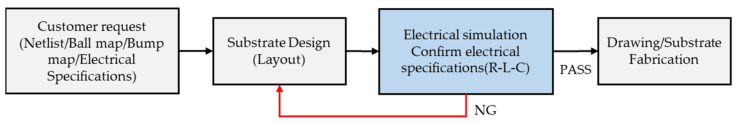
IC substrate generation process.

In the process of IC substrate generation, the substrate design is first performed according to the target circuit specification. The netlist of the corresponding circuit is associated with performing a post-layout simulation to verify the corresponding layout performance. If the post-layout simulation results are violated, the designer will adjust his layout and re-simulate. Figure 2 shows that this process is repeated until the simulated substrate design conforms to the RLC electrical specifications for interface transmission. The current process requires multiple simulation runs to meet the desired target circuit specification. Therefore, any inaccuracies in the design or components can produce misleading post-layout simulation results. Such misleading results can reduce yield and increase circuit design waste time.

**Figure 3:**
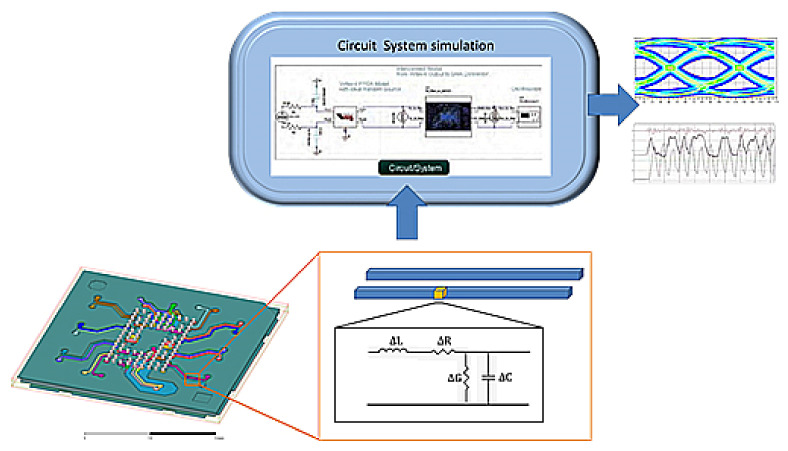
The illustration of RLC model system simulation and verification provided by SPIL (Siliconware Precision Industries Co., Ltd., Taichung City, Taiwan).

Signal transmission relies on the interconnected line group. According to the transmission line theory, the transmission line calibration model can replace the electrical characteristics of the signal and use an equivalent model. When the system simulation is based on the transmission line calibration model, the substrate RLC model, IC input/output buffer information specification (IBIS) model, and PCB electrical properties model are applied to the system simulations for system verification. The process is shown in Figure 3.

## 3. Forecasting RLC Values of Integrated Circuits by LSSVR-GA Models

### 3.1. LSSVR Models with Genetic Algorithms

The LSSVR method can be traced back to the SVM (Support Vector Machine) proposed by Cortes and Vapnik [22]. The SVM can handle classification and regression problems and performs better on small samples. Suykens and Vandewalle [23] proposed LSSVM. It solves the high computational burden problem of the SVM. The problem used to solve regression is called LSSVR [24].

Consider a given data set {$x_{i}$, $y_{i}$ | i = 1,2,3, …, n}, where $x_{i}\in R^{d}$ is the i^th^ input data including d features, and $y_{t}\in R$ is the i^th^ output data. Establishing the model for the LSSVR is as follows in Equation (1):

(1) $$y\;\left(x\right)=\omega^{T}\cdot \varphi \left(x\right)+b$$

where $\omega^{T}$ is the transposed form of the weight matrix, $\varphi \left(x\right)$ represents a nonlinear function that maps from the original dimensional feature space to a higher dimensional feature space, and b is a bias value.

The optimization problem to be solved by the model is presented as Equation (2):

(2) $$Min\;F\left(\omega ,\;e\right)=\frac{1}{2}\omega^{T}\omega +\frac{1}{2}\gamma \overset{n}{\underset{i=1}{\sum }}e_{i}^{2}\;Subject\;to:\;y_{i}=\omega^{T}\varphi \left(x_{i}\right)+b+e_{i}$$

where $F\left(\omega ,\;b\right)$ is lose function, γ is the regularization parameter, and e means the random error.

Because of the constraints, the optimal solution to the computational problem is very complicated. The Lagrange function is optimized and presented in Equation (3) to solve this problem:

(3) $$L\left(\omega ,\;b,\;e_{i},\;l_{i}\right)=F\left(\omega ,\;b\right)-\overset{n}{\underset{i=1}{\sum }}l_{i}(\;\omega^{T}\varphi \left(x_{i}\right)+b+e_{i}-y_{i})$$

where $L\left(\omega ,\;b,\;e_{t},\;l_{t}\right)$ is the Lagrange function and l is the Lagrange multiplier. After optimization using the KKT condition (Karush–Kuhn–Tucker condition), the formula is described in Equation (4)

(4) $$\frac{\partial L}{\partial \omega }=0\to \omega =\overset{n}{\underset{i=1}{\sum }}l_{i}\varphi \left(x_{i}\right)\;\frac{\partial L}{\partial b}=0\to \overset{n}{\underset{i=1}{\sum }}l_{i}=0\;\frac{\partial L}{\partial e_{i}}=0\to l_{i}=\gamma e_{i}\;\frac{\partial L}{\partial l_{i}}=0\to \;\omega^{T}\varphi \left(x_{i}\right)+b+e_{i}-y_{i}=0$$

The kernel function $K\left(x,x_{t}\right)$ is considered as follows in Equation (5):

(5) $$K\left(x,x_{i}\right)=\varphi {\left(x\right)}^{T}\varphi \left(x_{i}\right)$$

Finally, the model estimation formula by LSSVR can be obtained with Equation (6):

(6) $$y=\overset{n}{\underset{i=1}{\sum }}l_{i}K\left(x,x_{i}\right)+b$$

There are common kernel functions such as the string kernel [25], the radial basis function kernel (RBF) [26], and the polynomial kernel [27]. This study used the RBF kernel function in Equation (7), and the RBF kernel utilizes high-dimensional nonlinear mapping to resolve the nonlinear relationship between dependent and independent variables. The RBF kernel learned more complex decision boundaries:

(7) $$K\left(x,x_{i}\right)=\exp(-\frac{\|x-x_{i}{\|}^{2}}{2\sigma^{2}})$$

where σ is the parameter of the RBF kernel function. The decision of these two parameters—γ and σ—would affect the accuracy of the LSSVR model, so GA was performed to optimize these two parameters. The complete concept of GA was advocated by John Holland [28,29]. GA simulates the natural evolution law of natural ecology, imitates the survival of the fittest in the natural group, eliminates the inferior, and converges into a balanced mechanism under repeated iterations. GA is a search method used to solve optimization problems. Genes can select, crossover, and mutate. Better genes are passed to the new generation, and the inferior genes will be eliminated gradually. GA has been widely applied in solving optimization problems, data searches, artificial intelligence, and machine learning.

### 3.2. LSSVR-GA Architecture for RLC Prediction

**Figure 4:**
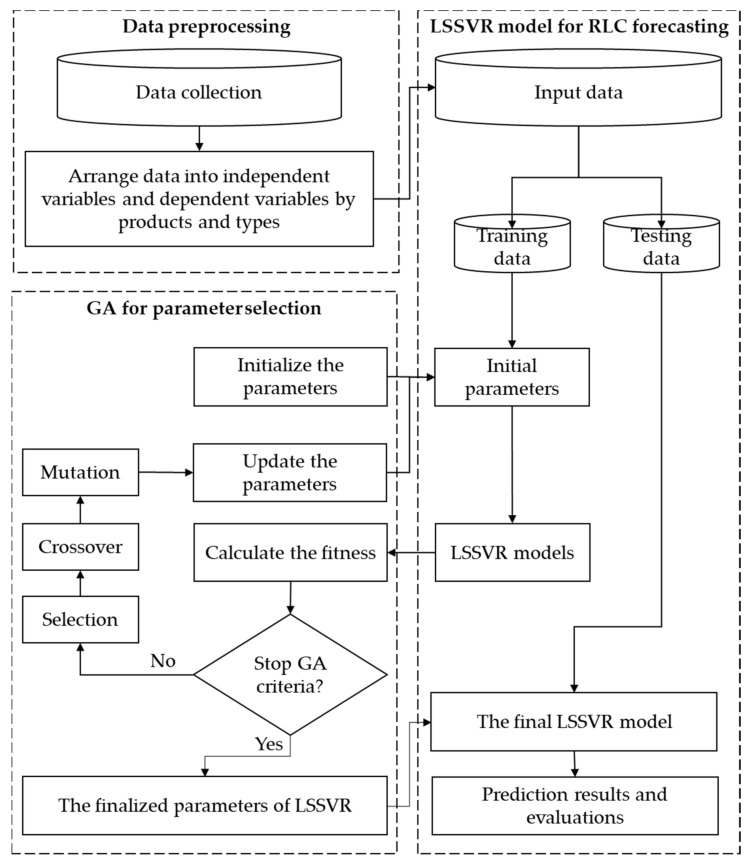
The framework of LSSVR-GA model for RLC values prediction.

Figure 4 illustrates the framework of the LSSVR-GA model in RLC (resistance, inductance, and capacitance) forecasting. It consists of 3 modules: data preprocessing, GA for parameter selection, and the LSSVR model for RLC forecasting. To solve the time-consuming problem of calculating the parameters for complex simulation software while verifying substrate designs, this study proposed a machine learning method to predict the RLC values for different product types.

The experimental data were semi-structured historical data provided by SPIL (Siliconware Precision Industries Co., Ltd.), including two different IC package process products, FC and WB. The historical data of different products of two-layer, four-layer, and six-layer PCB were selected based on these two processes. The three dependent variables for each product are resistance (R), inductance (L), and capacitance (C). The independent variables (X1~Xn) include ball, bump, base, L1, L2, L3, L4, L5, via, and wire. In the data preprocessing stage, this study integrated these scattered semi-structured data into one-to-one corresponding structured data between dependent and independent variables according to product types. It filled the missing values with 0. Table 2 describes the features and samples in predicting RLC for different data sets of substrate products.

The preprocessed data sets were divided into 80% training data and 20% testing data. The training data were used to build the LSSVR model with the parameters optimized by GA. Before applying GA, it is necessary to encode the parameters to be optimized into a group of chromosomes. The common encoding methods include binary, real, multi-objective, parallel, chaotic, and hybrid GA [30]. Considering the simplicity of implementation for factory operators, this study used the binary-coded GA to optimize the parameters of LSSVR, and each digital bit represented a gene. The length of the chromosome was defined according to the spatial range of the actual problem to be solved. The real number represented by the binary encoded was calculated as Equation (8).

(8) $$RV=LB+\frac{\left(\sum_{i=1}^{l}d_{i}\cdot 2^{i-1}\right)}{2^{l}-1}\cdot \left(UB-LB\right)$$

where RV is the real number represented by the binary encoded, LB is the lower bound of the spatial range, UB is the upper bound of the spatial range, l is the encoded bit length, and $d_{i}$ is the bit value of the i^th^ bit.

**Figure 5:**
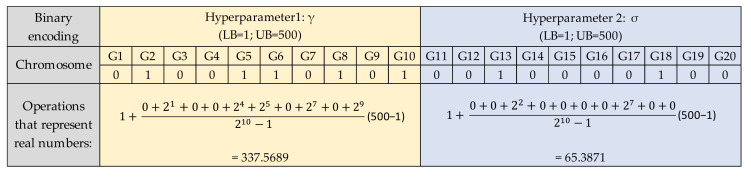
The LSSVR parameter encoding and operation of representing real numbers.

**Figure 6:**
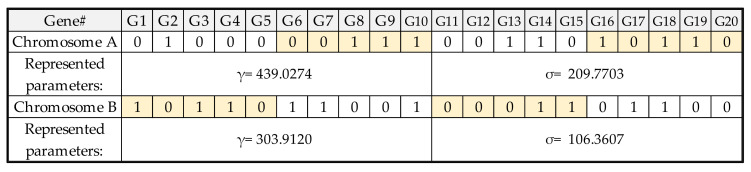
The binary encoding for parameters of LSSVR at the stage of initial population.

**Figure 7:**
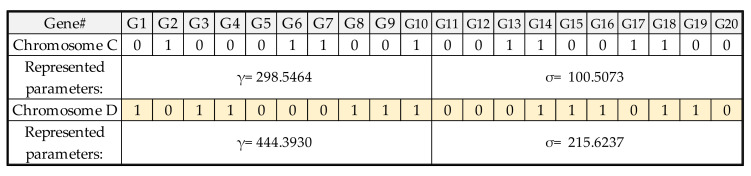
The binary encoding for parameters of LSSVR after multi-point crossover.

**Figure 8:**
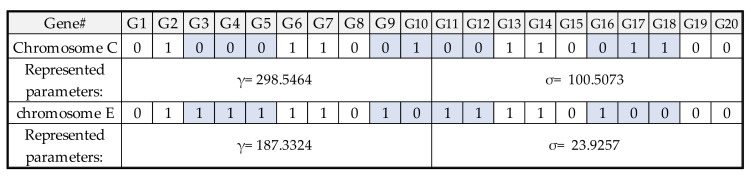
The binary encoding for parameters of LSSVR after mutation from chromosome C to E.

Figure 5 shows the LSSVR model’s encoded parameters—γ and σ—and the operation of real numbers. Each parameter consists of 10 genes, and the LSSVR model has two parameters. These two parameters represent 20 genes as a chromosome. The lower and upper bounds of the two parameters are both 1 and 500, and the real numbers represented are calculated accordingly. It is also necessary to define the optimized procedure settings of LSSVR-GA. The population size, iteration, crossover rate, and mutation rate were arranged at 40, 20, 0.8, and 0.1, respectively. When starting GA, the parameters must first be initialized as the input parameter of the LSSVR model. The training result of the LSSVR model is calculated by the fitness function. This is to evaluate the stopping conditions for GA. If conditions are not met, it will go through the process as in Figure 6, Figure 7 and Figure 8, and the GA selection-crossover-mutation process will have a new generation. Good chromosomes have more opportunities to be selected. Unfit and less fit chromosomes are gradually eliminated. Therefore, the updated parameters are used as input parameters of the LSSVR model. To find the best-fit parameters of LSSVR, repeat the fitness function to compute the evaluation until the GA stop condition. Then, set the best-fit parameters of LSSVR in the final LSSVR model and perform RLC predictions. 

## 4. Numerical Results

Predicted results are evaluated and analyzed with the testing data to examine the effectiveness and interpretability of the proposed method. The evaluation is measured by mean absolute percentage error (MAPE (%)), weighted absolute percent error measure (WAPE (%)), and normalized mean absolute error (NMAE), as shown in Equations (9)–(11).

(9) $$MAPE\left(\%\right)=\frac{100}{n}\overset{n}{\underset{i=1}{\sum }}\left|\frac{{\hat{Y}}_{i}-Y_{i}}{Y_{i}}\right|\;$$

(10) $$WAPE\left(\%\right)=100\frac{\sum_{i=1}^{n}\left|{\hat{Y}}_{i}-Y_{i}\right|}{\sum_{i=1}^{n}Y_{i}}$$

(11) $$NMAE=\frac{1}{max\;Y-minY}\;\left(\frac{1}{n}\sum_{i=1}^{n}\left|{\hat{Y}}_{i}-Y_{i}\right|\right)$$

where ${\hat{Y}}_{i}$ is the i^th^ predict value, $Y_{i}$ is the i^th^ actual value, and i = 1~n.

Three other forecasting models with genetic algorithms, namely backpropagation neural networks (BPNN-GA), random forest (RF-GA), and eXtreme gradient boosting (XGBoost-GA), were employed to deal with the same data. Table 3 illustrates parameters determined by genetic algorithms to predict LCR values of different forecasting models. Lewis [31] reported that forecasting performance measured by MAPE values could be depicted in Table 4. Table 5 lists the MAPE, WAPE, and NMAE values of the four forecasting models. 

The average performance of these six products is at the levels of good or highly accurate in predicting RLC using LSSVR-GA models. Furthermore, the LSSVR-GA models can generate average more accurate results than the other three forecasting models in terms of MAPE, WAPE, and NMAE. 

## 5. Conclusions

This study outlines an efficient method for predicting RLC circuit simulation in the IC package process using LSSVR-GA hybrid models. This method can be used to predict the integrity of RLC circuits. The ability to accurately predict analog circuits is essential to the IC package industry due to the timesaving in substrate design and process optimization. The numerical results revealed that the designed LSSVR-GA method is a feasible, effective, and efficient alternative for forecasting RLC values. For future research, one potential direction is to employ deep learning approaches to cope with the same data sets used in this study to improve forecasting accuracy. The other possible direction is to apply the presented LSSVR-GA framework to more complex circuit cases to examine performance.

## Figures and Tables

**Table 2:** The features and samples in predicting RLC for different substrate products.

Data Sets	Features (X Variables)											Samples
	Ball	Bump	Base	L1	L2	L3	L4	L5	Via	Wire	Total	
FC_2L_T1	1	1	2	2					2		8 X	2232
FC_4L_T1	1	1	2	2	2	2			6		16 X	999
FC_6L_T1	1	1	2	2	2	2	2	2	10		24 X	742
WB_2L_T1	1		2	2					2	1	8 X	1400
WB_4L_T1	1		2	2	2	2			6	1	16 X	2704
WB_6L_T1	1		2	2	2	2	2	2	10	1	24 X	450

**Table 3:** Parameters of forecasting models provided by genetic algorithms in LCR values prediction.

Method	Parameters	Ls (nH)	Cs (pF)	R (mΩ)	Ls (nH)	Cs (pF)	R (mΩ)	Ls (nH)	Cs (pF)	R (mΩ)
Products		FC_2L_T1			FC_4L_T1			FC_6L_T1		
GA-LSSVR	gamma	3.5241	220.2197	266.8764	386.9266	388.4704	336.2926	237.4034	386.9266	199.8153
	sigma	490.8269	2.3006	1.7176	1.4692	3.2476	4.0466	2.1650	1.4692	1.0638
GA-BPNN	learning rate	0.278163	0.725969	0.380188	0.316992	0.77501	0.668117	0.609052	0.168028	0.1674687
	momentum	0.790603	0.415267	0.424905	0.803841	0.748888	0.770531	0.864727	0.890341	0.186707
GA-RF	NTG *	162	122	313	281	268	423	491	316	159
	MTRY *	7	7	7	16	14	15	22	24	23
	NS *	32	25	13	9	11	9	10	5	11
	SSD *	3	5	6	7	15	12	23	16	23
	MN *	99	99	99	99	90	94	100	95	90
GA-XGBoost	CB *	0.9652	0.95019	0.94883	0.88056	0.85548	0.97795	0.83369	0.83964	0.95994
	SS *	0.73781	0.95313	0.9539	0.93509	0.93643	0.9374	0.9856	0.93392	0.9727
	MD *	9	10	9	9	9	9	10	9	7
	learning rate	0.0792	0.0981	0.08608	0.08813	0.08183	0.09519	0.04556	0.07721	0.07101
	gamma	0.03703	0.44082	0.9867	0.26736	0.00473	0.99912	0.0118	0.00621	0.19647
	MW *	4.67495	3.0224	3.60389	3.51156	3.60387	3.88513	3.15073	5.40802	3.43816
	Lambda *	0.84143	1.0274	0.73609	0.62597	1.06227	0.66675	0.58733	1.23425	0.66977
**Products**		**WB_2L_T1**			**WB_4L_T1**			**WB_6L_T1**		
GA-LSSVR	gamma	487.8397	461.7180	253.7229	260.8126	138.0979	291.6471	264.8891	386.9266	261.5888
	sigma	2.1064	1.6639	3.5726	2.1926	1.6762	2.3001	2.0693	1.4692	4.8404
GA-BPNN	learning rate	0.195829	0.558181	0.897148	0.270761	0.652679	0.405238	0.301158	0.86155	0.105814
	momentum	0.864213	0.65859	0.694771	0.894708	0.765861	0.88196	0.318845	0.639695	0.634643
GA-RF	NTG *	338	179	356	275	435	193	492	381	255
	MTRY *	7	7	7	14	11	13	22	24	21
	NS *	21	16	20	33	29	32	8	4	8
	SSD *	7	5	4	5	11	6	5	7	18
	MN *	97	100	99	99	100	98	99	96	85
GA-XGBoost	CB *	0.91655	0.88732	0.87073	0.98352	0.94273	0.82735	0.90711	0.81666	0.94269
	SS *	0.52606	0.70242	0.92529	0.66074	0.89115	0.91367	0.74513	0.90611	0.9466
	MD *	10	8	10	9	9	10	10	8	9
	learning rate	0.09689	0.06631	0.08815	0.09564	0.08211	0.08766	0.09008	0.09676	0.08981
	gamma	0.00929	0.02931	0.11065	0.01612	0.00016	0.1768	0.01459	0.01058	0.48457
	MW *	3.52074	5.63888	3.38181	4.18612	4.3746	3.62682	3.1284	3.44193	4.72415
	Lambda *	0.56806	1.17406	0.87922	0.78879	0.77885	0.52211	0.7232	1.13934	0.79814

* NTG: the number of trees to grow; MTRY: the number of variables used at each split; NS: the minimum size of terminal nodes; SSD: the sample sizes to draw; MN: the maximum number of terminal nodes trees in the forest can have; CB: subsample percentage of columns while generating new trees; SS: the subsample ration of training cases; MD: the maximum depth of the tree; MW: the minimum sum of weights related to child nodes; lambda: the L2 regularization term of weights.

**Table 4:** Levels of forecasting accuracy measured by MAPE [31].

MAPE Values (%)	Accuracy
<10	Highly accurate prediction
10–20	Good prediction
20–50	Reasonable prediction
>50	Inaccurate prediction

**Table 5:** Forecasting results of different models in terms of MAPE, WAPE, and NMAE.

Product Type	Method	Ls (nH)			Cs (pF)			R (mΩ)		
		MAPE (%)	WAPE (%)	NMAE	MAPE (%)	WAPE (%)	NMAE	MAPE (%)	WAPE (%)	NMAE
FC_2L_T1	GA-LSSVR	**16.88**	**15.63**	**0.04818**	**13.25**	**13.47**	**0.05634**	**6.20**	**6.48**	**0.02528**
	GA-BPNN	18.03	17.55	0.05410	13.93	13.49	0.05642	7.48	6.72	0.02623
	GA-RF	18.64	18.79	0.05792	13.43	13.44	0.05619	7.25	6.96	0.02712
	GA-XGBoost	19.01	19.49	0.06008	14.12	14.02	0.05862	8.74	7.77	0.03029
FC_4L_T1	GA-LSSVR	**12.18**	**8.92**	**0.01625**	**6.75**	**5.11**	**0.01527**	**12.15**	**5.99**	**0.00845**
	GA-BPNN	35.86	22.67	0.04130	9.81	8.12	0.02423	25.05	14.35	0.02024
	GA-RF	12.30	9.61	0.01751	8.60	6.95	0.02074	16.68	7.80	0.01100
	GA-XGBoost	15.16	11.06	0.02015	7.54	6.01	0.01796	17.85	7.87	0.01110
FC_6L_T1	GA-LSSVR	**10.35**	**7.81**	**0.05383**	**9.09**	**7.42**	**0.04804**	**11.99**	**8.33**	**0.04320**
	GA-BPNN	10.37	8.74	0.06019	9.26	8.63	0.05588	15.77	12.87	0.06674
	GA-RF	10.40	8.56	0.05897	9.46	8.35	0.05402	12.10	9.74	0.05053
	GA-XGBoost	10.87	9.15	0.06302	9.32	8.27	0.05352	12.23	10.45	0.05420
WB_2L_T1	GA-LSSVR	**13.28**	**12.63**	**0.05518**	**5.61**	**5.25**	**0.02018**	**7.21**	**6.79**	**0.02781**
	GA-BPNN	15.23	13.39	0.05852	6.64	6.00	0.02307	11.40	9.71	0.03980
	GA-RF	13.86	12.40	0.05417	6.10	5.86	0.02255	9.70	8.98	0.03680
	GA-XGBoost	13.42	11.48	0.05015	6.62	6.07	0.02337	9.05	8.05	0.03298
WB_4L_T1	GA-LSSVR	**14.54**	**11.65**	**0.03301**	**6.71**	**5.84**	**0.02074**	**10.00**	**7.10**	**0.02697**
	GA-BPNN	16.00	12.19	0.03453	9.19	7.53	0.02676	13.19	9.20	0.03497
	GA-RF	14.96	12.28	0.03480	9.16	7.79	0.02767	10.59	8.08	0.03071
	GA-XGBoost	15.81	11.74	0.03325	7.33	6.35	0.02255	10.10	7.47	0.02840
WB_6L_T1	GA-LSSVR	**8.68**	**9.28**	**0.05880**	**6.48**	**7.31**	**0.03904**	**4.08**	**4.89**	**0.03481**
	GA-BPNN	9.95	9.21	0.05838	6.56	6.63	0.03541	4.36	4.63	0.03299
	GA-RF	10.13	9.92	0.06289	6.61	6.79	0.03624	7.39	8.18	0.05819
	GA-XGBoost	9.60	8.75	0.05546	6.60	6.73	0.03593	6.71	7.43	0.05285
**Average**	GA-LSSVR	**12.65**	**10.99**	**0.04421**	**7.98**	**7.40**	**0.03327**	**8.60**	**6.60**	**0.02775**
	GA-BPNN	17.57	13.96	0.05117	9.23	8.40	0.03696	12.87	9.58	0.03683
	GA-RF	13.38	11.93	0.04771	8.89	8.19	0.03624	10.62	8.29	0.03573
	GA-XGBoost	13.98	11.94	0.04702	8.59	7.91	0.03533	10.78	8.17	0.03497

## Data Availability

Not applicable.
